# Nursing Care Using Gordon’s Model in Allogeneic Stem Cell Transplantation for Acute Myeloid Leukemia: A Case Report

**DOI:** 10.3390/healthcare14213777

**Published:** 2026-11-05

**Authors:** Gamze Oğuz

**Affiliations:** Department of Nursing, Faculty of Health Sciences, Atlas University, Istanbul 34408, Türkiye; gamze.oguz@atlas.edu.tr

**Keywords:** hematopoietic stem cell transplantation, acute myeloid leukemia, nursing care, oncology nursing, stomatitis, case reports

## Abstract

**Highlights:**

**What are the main findings?**

- Gordon’s 11 Functional Health Patterns were systematically applied, pattern by pattern, throughout an allogeneic HSCT course for acute myeloid leukemia, with a day-level clinical timeline and NANDA-I–NIC–NOC alignment.
- Outcome tracking with the nurse-rated Beck Oral Assessment Scale and the patient-reported Numeric Rating Scale showed resolution of oral mucositis and neutrophil engraftment by day +16, alongside systematic attention to the psychosocial needs arising from the patient’s prior BRCA-related mastectomy.

**What are the implications of the main findings?**

- Gordon’s model provided a practical, holistic template for organizing nursing assessment and care planning in this case of allo-HSCT.
- Attending explicitly to a patient’s layered oncologic history alongside acute transplant complications may help nurses individualize psychosocial care during allo-HSCT.

**Abstract:**

Background: Allogeneic hematopoietic stem cell transplantation (allo-HSCT) exposes patients to complex physiological complications such as oral mucositis, nutritional deficits, infection, and graft-versus-host disease, alongside profound psychosocial distress, demanding structured, evidence-based nursing approaches. While Gordon’s functional health patterns model is used in oncology nursing, it has been described in only a few HSCT case reports, and few report outcomes with defined measures. Case Summary: A 38-year-old Turkish woman with newly diagnosed acute myeloid leukemia (AML-M5) underwent allo-HSCT from an HLA-matched sibling donor after fludarabine/busulfan myeloablative conditioning. Nursing assessment and care planning were organized around Gordon’s 11 Functional Health Patterns. Priority NANDA-I diagnoses were impaired oral mucous membrane integrity with acute pain (oral mucositis), inadequate nutritional intake, risk for infection (neutropenia), inadequate health knowledge, risk for imbalanced blood pressure (orthostatic hypotension), excessive anxiety, and excessive parental role conflict; interventions were mapped to NIC and outcomes were mapped to NOC outcome labels (indicator ratings were not recorded). Oral mucositis was documented with a Beck Oral Assessment Scale (BOAS) score of 15 on day +7, progressed to grade 3 by day +9, and required topical anesthetics, intensive oral care (saline rinses from day +4), and nutritional support; the BOAS score fell to 5 by day +14. Neutrophil engraftment occurred on day +16 without granulocyte colony-stimulating factor, and the patient was discharged on day +18 (3 March 2023) with comprehensive self-management education. Anxiety related to her prior BRCA-positive mastectomy, together with concerns about fertility and family roles, was addressed within this framework; psychosocial outcomes rest on clinical observation and patient report. Conclusion: In this case, Gordon’s model was useful for structuring comprehensive nursing assessment and care planning during allo-HSCT by bringing physiological and psychosocial domains into a single care plan; as a single case, it cannot show that the model itself influenced the clinical outcomes.

## 1. Introduction

Acute myeloid leukemia (AML) is a heterogeneous malignancy characterized by the uncontrolled proliferation of myeloid cells in the bone marrow and peripheral blood, ultimately disrupting normal hematopoiesis and giving rise to life-threatening cytopenias. AML is classified using two systems. The older French–American–British (FAB) classification, largely superseded in routine clinical practice, is still used to describe morphologic subtypes such as acute monocytic leukemia (M5), the subtype in the present case. The World Health Organization (WHO) classification, now the standard, instead categorizes AML by biological features, including recurrent genetic abnormalities, myelodysplasia-related changes (AML-MR in the 2022 edition), and therapy-related myeloid neoplasms (t-MN). AML is estimated to account for approximately 15–20% of all acute leukemia cases in the pediatric population, compared with roughly 80% in adults [1,2]. The 5-year relative survival rate for patients diagnosed with acute myeloid leukemia in the US is currently 30.5%, compared with just 18% in 2000. Improvements in AML outcomes are attributable to numerous factors, including advances in best supportive care (e.g., antifungal and antibacterial prophylaxis and treatment), a reduction in transplant-related mortality that has allowed more patients—including older patients—to benefit from this curative approach, and greater availability of effective, novel treatment regimens [3]. For the majority of AML patients, allogeneic hematopoietic stem cell transplantation (allo-HSCT) remains the only treatment with curative potential; its curative effect relies primarily on the graft-versus-leukemia (GvL) reaction mediated by transplanted immune cells, chiefly T cells and NK cells. For this reason, allo-HSCT is regarded as the prototype of cellular immunotherapy. Nevertheless, relapse following allo-HSCT is common and is associated with a poor prognosis; this indicates that, despite the initial control exerted by the allogeneic immune system, leukemic cells develop escape strategies over time [2]. Patients undergoing hematopoietic stem cell transplantation (HSCT) face both physical and psychological challenges. Common complications following HSCT include infections, gastrointestinal disturbances, pain, fatigue, allergic reactions, and graft-versus-host disease. Late complications of HSCT can affect multiple organ systems, including the eyes, heart, lungs, endocrine organs, and bones [4,5]. With respect to psychological effects, fear, distress, and isolation significantly affect patients undergoing HSCT both socially and emotionally, undermining their quality of life. Beyond these physical and psychological challenges, these patients may also contend with additional burdens such as referrals, treatments, hospitalization, transportation, financial strain, absenteeism from work, and disrupted social relationships. Furthermore, these patients experience lifestyle changes, including reduced physical activity, owing to treatments that compromise their muscle strength and endurance [6,7].

Gordon’s Functional Health Patterns (FHP) model enables the systematic collection, categorization, and standardization of patient data. Through Gordon’s nursing model, individuals are holistically assessed from a biopsychosocial perspective within the framework of 11 functional health pattern categories [8,9,10]. The purpose of this case report is to present nursing care based on Gordon’s FHP Model and the North American Nursing Diagnosis Association-International (NANDA-I) Taxonomy II classification system for a 38-year-old patient diagnosed with AML who underwent allogeneic hematopoietic stem cell transplantation (allo-HSCT).

The case is notable for the patient’s complex psychosocial profile; her prior history of triple-negative breast cancer and bilateral mastectomy due to a BRCA mutation was associated with intense symptoms such as altered body image, fertility-related concerns, and family role strain, necessitating targeted nursing interventions. This report adds to the few existing Gordon-based nursing case reports in HSCT [11] by presenting a model-driven nursing assessment, NANDA–NIC–NOC-aligned interventions, and a day-by-day account of measurable patient outcomes, thereby illustrating how a structured framework can be applied in this clinical context.

## 2. Case Presentation

### 2.1. Chief Complaints

A 38-year-old woman presented to the clinic with complaints of fatigue, dizziness, spontaneous bruising, exertional dyspnea, and bone pain.

### 2.2. History of Present Illness

A 38-year-old female patient presented to the clinic with complaints of fatigue, dizziness, spontaneous bruising, exertional dyspnea, and bone pain. On initial evaluation, complete blood count results dated 3 November 2022 revealed markedly abnormal values, with a hemoglobin level of 8 g/dL and a platelet count of 55,000/µL; the leukocyte count was 6800/µL, the absolute neutrophil, lymphocyte, and monocyte counts were 1.12, 3.05, and 2.62 × 10^3^/µL, respectively (monocytosis). In addition, the LDH level was found to be above the reference range at 317 U/L, indicating increased cellular turnover and raising suspicion for an underlying hematologic malignancy. Given the concurrent findings of anemia, thrombocytopenia, monocytosis, and elevated LDH, bone marrow flow cytometry was performed on 9 November 2022 for further investigation; 77% of all cells were found to be of monoblastic lineage, consistent with a diagnosis of AML-M5. Fluorescence in situ hybridization (FISH) analysis revealed NUP98 positivity at 82%, while 3q26, 5q31, t(6;9), 7q22, t(7;12), t(8;21), t(9;22), 11q23, t(15;17), 16q22, and TP53 (17p13) were negative. Karyotype analysis yielded the result 46,XX,inv(11)(p15q23) [12] /46,XX [3] (as reported by the cytogenetics laboratory; the partner gene and the FISH probe design were not stated in the report). The breakpoint usually described for the NUP98::DDX10 fusion is inv(11)(p15q22) [13], whereas the report gives band q23; we reproduce the laboratory’s description and cannot resolve this from the available records. In light of these findings, the patient’s clinical presentation, laboratory results, and immunophenotypic characteristics were evaluated as a whole, leading to a diagnosis of NUP98-positive AML-M5. In the 2022 WHO and International Consensus classifications, AML with NUP98 rearrangement is recognized as a defined genetic subtype [14,15]; molecular panel results (e.g., FLT3, NPM1) and European LeukemiaNet 2022 risk stratification are not available in the documentation used for this report. At transplantation, the indication was recorded as AML in first remission, intermediate risk.

The patient received induction chemotherapy according to the 3 + 7 protocol between 15 November and 15 December 2022. A bone marrow biopsy performed on 14 December 2022, was consistent with remission, and the NUP98 value reported on 12 December 2022, was 6%. The record does not state the method; the baseline value (82%) was obtained by FISH, which is not a standard or sensitive method for measurable residual disease (MRD). No pre-transplant MRD assessment, such as flow cytometry, or marrow evaluation after consolidation is documented in the available records. As part of consolidation therapy, the HIDAC (high-dose cytarabine) protocol was administered between 2 and 7 January 2023. On 6 February 2023, the patient was admitted to the clinic for allogeneic peripheral blood stem cell transplantation from her fully HLA-matched sister.

### 2.3. History of Past Illness

Upon review of the patient’s medical history, it was noted that she had been diagnosed with triple-negative breast cancer in 2021 and, in addition to this diagnosis, had been under regular follow-up for Hashimoto’s thyroiditis for 10 years. As part of the COVID-19 vaccination program, the patient had received a total of 5 vaccine doses; specifically, 2 doses of Sinovac followed by 3 doses of BioNTech (BNT), with her most recent vaccination administered in March 2022. Furthermore, because of a BRCA1 gene mutation, she had undergone bilateral mastectomy and had a history of bilateral breast implants following this procedure. Details of the treatment she received for the breast cancer are not documented in the available records.

### 2.4. Personal and Family History

The patient’s history revealed no tobacco or alcohol use, and there were no known drug allergies. Her obstetric history was G1P1A0, with one healthy child, currently 4 years of age. The family history was unremarkable for hematologic malignancy. Her father, aged 74, was living and had a history of coronary artery bypass surgery. Her mother, aged 62, was also living. The patient had a 41-year-old sister who was married and in good health. The family history was notable for lung cancer in paternal uncles.

### 2.5. Physical Examination

On examination, the patient was conscious, oriented, and cooperative. Vital signs were recorded as follows (blood pressure, 119/63 mmHg; pulse, 116 bpm; oxygen saturation, 97%; temperature, 36.6 °C; initial evaluation, 12 November 2022). She appeared pale, and no petechiae, purpura, or hematoma were noted. Lymphadenopathy or organomegaly was not observed. The results of cardiopulmonary and neurological examinations were unremarkable. Functional status was good (Karnofsky Performance Score, 90). Body mass index was 19.9 kg/m^2^ (height 172 cm, weight 59 kg).

### 2.6. Laboratory Examinations

Pretransplant evaluation demonstrated preserved organ function. The hematopoietic cell transplantation comorbidity index was recorded as 4 (depression, 1; prior solid tumor, 3). The donor was her 41-year-old HLA 6/6-matched sister (ABO A Rh-positive, as in the recipient; CMV IgG positive in both). Echocardiography showed an ejection fraction of 55–60%, pulmonary function tests revealed an FEV1 of 114%, and normal renal function was confirmed (glomerular filtration rate 139.49 mL/min). Ferritin was 855 ng/mL; thyroid-stimulating hormone was low (0.28 µIU/mL), leading to levothyroxine dose adjustment. Quantiferon-TB was positive, and isoniazid prophylaxis was initiated. HSV-1 and HSV-2 IgM were positive and acyclovir prophylaxis was initiated. The baseline laboratory findings are summarized in Table 1.

### 2.7. Imaging Examinations

Pretransplant abdominal and thoracic CT (2 February 2023) showed no evidence of active infection or malignancy. Abdominal CT was unremarkable, with normal liver, biliary tree, spleen, pancreas, adrenal glands, kidneys, and pelvic organs. Thoracic CT confirmed correct positioning of the chemotherapy port and central venous catheters and bilateral breast implants; incidental findings included a 3-mm nonspecific right upper lobe nodule, calcifications in the left upper lobe, and a minimal pericardial effusion, none of which required intervention.

### 2.8. Final Diagnosis

The patient, diagnosed with AML-M5, received induction chemotherapy according to the 3 + 7 protocol between 15 November and 15 December 2022. A bone marrow biopsy performed on 14 December 2022, was consistent with remission, and the NUP98 value reported on 12 December 2022, was 6% (the method is not stated in the record; see Section 2.2). As part of consolidation therapy, the HIDAC (high-dose cytarabine) protocol was administered between 2 and 7 January 2023. On 6 February 2023, the patient was admitted to the clinic for allogeneic peripheral blood stem cell transplantation from her fully HLA-matched sister.

### 2.9. Treatment and Clinical Course

Before allogeneic peripheral blood stem cell transplantation (13 February 2023), the patient received fludarabine and busulfan as the conditioning regimen; she also received cyclosporine A (CSA), methotrexate (MTX), and mycophenolate mofetil (MMF) for GVHD prophylaxis. The transplant course was complicated by grade 3 oral mucositis; neutrophil engraftment was nevertheless achieved on day +16 without the use of G-CSF. During follow-up, the patient’s general condition remained stable, oropharyngeal findings gradually improved, and lung sounds and abdominal examination remained within normal limits. Platelet transfusion was performed prophylactically before counts fell below 20,000/µL, with two apheresis platelet suspensions and one packed red blood cell suspension transfused during the course. Empiric meropenem was initiated during the neutropenic period; as the blood culture obtained on 25 February 2023 showed no growth and CRP values declined, treatment was discontinued after recovery from neutropenia. During hospitalization, the subclavian central venous catheter was removed, and an episode of orthostatic hypotension (likely engraftment-related) developed, which was successfully managed with methylprednisolone 2 × 40 mg and mobilization restriction. A urine culture was obtained because of urinary tract complaints, with no notable findings on urinalysis. Following clinical stabilization, the patient was discharged on 3 March 2023 (day +18), and a follow-up visit was scheduled for 8 March 2023 (day +23).

Myeloablative conditioning began on day −6 (7 February 2023): fludarabine 30 mg/m^2^/day on days −6 to −2 and intravenous busulfan 3.2 mg/kg/day on days −6 to −3. GVHD prophylaxis consisted of cyclosporine A (3 mg/kg starting day −1), short-course methotrexate (15 mg/m^2^ day +1, 10 mg/m^2^ days +3 and +6), and mycophenolate mofetil (15 mg/kg twice daily, days +1 to +28).

On 13 February 2023 (day 0), allogeneic peripheral blood stem cell transplantation was uneventfully completed. The graft volume was 330 mL, providing a CD34+ cell dose of 4.66 × 10^6^/kg, total nucleated cell count 10.85 × 10^8^/kg, mononuclear cell count 5.31 × 10^8^/kg, and CD3+ T-cell count 2.69 × 10^8^/kg. Cell viability was 99.89%, and the product was infused fresh. The clinical course from conditioning to discharge is summarized in Table 2.

### 2.10. Data Sources and Nursing Assessment Approach

This case report follows the CARE reporting guideline. The case description is based on the patient’s hospital epicrisis and clinical records. Nursing care was organized according to Gordon’s 11 Functional Health Patterns; diagnoses were selected from NANDA-I 2024–2026, interventions from NIC, and outcomes from NOC [8,16,17]. Mucositis was assessed with the Beck Oral Assessment Scale (BOAS; nursing assessment) and pain with the Numeric Rating Scale (NRS; patient-reported); the physician-recorded clinical mucositis grade is reported separately, and the grading scale is not specified in the record. Priority diagnoses (Section 4.1) were those that corresponded to documented clinical events requiring intervention or to documented education and psychosocial needs. NOC was used as a classification framework, not as a quantitative outcome measurement system; indicator ratings were not recorded, so outcomes are described using measured clinical parameters, clinical observation, or patient report, and the type of evidence is stated for each. Physician-documented information (for example, cytogenetic and molecular results, measurable residual disease, donor evaluation, treatment of the earlier breast cancer, and prescribing rationale) is reported as it appears in the medical record; the physician reports and their detailed results are outside the scope of the nursing care plan and are not included in this report. The patient perspective in Section 4.2 was obtained during an individual interview at the follow-up visit one week after discharge (8 March 2023, day +23). The interview was conducted in Turkish, and the patient’s statements were conveyed in the report in English translation. The patient gave her consent to including her account in this case report.

## 3. Nursing Care Structured According to Gordon’s Functional Health Patterns

Each pattern is summarized below with respect to its assessment focal points, priority NANDA-I diagnoses, NIC interventions aligned with key implementation details, and NOC outcome labels [16,17] (NOC is used here as a classification framework; no NOC ratings were recorded). Table 3 presents a comprehensive NANDA-NIC-NOC linkage map for all patterns; Section 4.1 links the priority diagnoses to assessment findings, interventions, and observed outcomes.

### 3.1. Health Perception–Health Management

At admission, the patient demonstrated a basic understanding of the AML diagnosis and the indications for transplantation; however, her knowledge of the conditioning regimen, the GVHD prophylaxis protocol, neutropenic precautions, and post-discharge self-management was inadequate. This knowledge deficit was clinically significant, as adherence to complex medication schedules—including cyclosporine A with therapeutic drug monitoring, antifungal and antiviral prophylaxis, and isoniazid for latent tuberculosis—directly affects transplantation outcomes. Structured education was provided covering the conditioning protocol, the GVHD prophylaxis schedule, the purpose and adverse effects of each medication, the necessity of therapeutic drug monitoring, and infection prevention behaviors. This education was reinforced at transitions of care, and the teach-back method was used to verify comprehension.

NANDA-I: Risk for infection; inadequate health knowledge (transplant process, infection prevention); readiness for enhanced health self-management.

NIC: Teaching: procedure/treatment; medication management; infection protection.

NOC: Knowledge: infection management, health-seeking behavior; infection severity [18].

### 3.2. Nutritional–Metabolic

Before conditioning, the patient had a BMI of 19.9 kg/m^2^ with adequate oral intake and normal renal and hepatic function. On day +7, progressive oropharyngeal pain and dysphagia developed. Mucositis severity was documented with the Beck Oral Assessment Scale (BOAS; score 15 on day +7, when the physician recorded clinical grade 2) and progressed to clinical grade 3 on day +9, with severe dysphagia limiting oral intake [19].

The oral medications were converted to intravenous formulations on day +9. Enteral nutritional supplements were initiated, and transdermal opioid analgesia (fentanyl patch 25 µg/h) was administered on day +9. Antifungal prophylaxis was switched from posaconazole to the intravenous administration of micafungin. Daily weight, intake–output, and serum electrolyte levels were monitored. By day +14–15, the oropharyngeal mucosa improved markedly, and maintenance intravenous fluids were stopped on day +12.

NANDA-I: Impaired oral mucous membrane integrity; inadequate nutritional intake; risk for impaired water-electrolyte balance.

NIC: Oral health restoration; nutrition management; pain management: acute; electrolyte management.

NOC: Oral mucous membrane integrity; nutritional status: food and fluid intake; pain control; hydration.

### 3.3. Elimination

Baseline renal function was within normal limits, and the patient reported no bowel or urinary complaints during the conditioning process. On day +9, hemorrhoidal bleeding developed; this was managed with single-donor apheresis platelet transfusion and local supportive care. Nurses closely monitored the frequency of bleeding, reinforced perianal hygiene, and emphasized to the patient the need to avoid straining during defecation. On day +15, the patient reported dysuria and genital burning; urinalysis and culture studies revealed no significant pathology. Fluid balance was meticulously monitored. Urinalysis showed no notable findings (no hematuria was documented), so hemorrhagic cystitis was not apparent. In this setting, genital burning with a negative urinalysis and culture may also reflect conditioning-related mucosal toxicity, candidal or herpes simplex involvement (HSV IgM was positive before transplantation), or, had urinary symptoms persisted, BK virus-associated cystitis; these possibilities fall within physician evaluation, although genitourinary complications of allogeneic HSCT are well recognized [20,21]. For this reason, structured nursing surveillance, timely reporting of symptoms, and early multidisciplinary evaluation remain indispensable components of nursing care during this phase, even when initial findings are reassuring [20].

NANDA-I: Risk for excessive bleeding; risk for impaired intestinal elimination; impaired tissue integrity (perianal); impaired urinary elimination.

NIC: Bowel management; bleeding precautions; bleeding reduction; urinary elimination management; fluid management.

NOC: Elimination status: bowel; elimination status: urinary; bleeding severity; fluid balance.

### 3.4. Activity–Exercise

The patient was admitted with good functional capacity (Karnofsky score 90). Throughout the conditioning process and the early post-transplantation period, the patient remained hemodynamically stable and independently performed basic activities of daily living within the framework of neutropenic precautions. In-room mobilization was encouraged to prevent deconditioning. On day +16, orthostatic hypotension was confirmed (supine blood pressure of 110/69 mmHg dropping to 77/42 mmHg upon standing); this was attributed to hemodynamic changes associated with engraftment. Isotonic fluid infusion and methylprednisolone (2 × 40 mg) were administered, and fall prevention measures were implemented. The episode is labeled risk for imbalanced blood pressure (00362) and not risk for decreased cardiac output (00240): no cardiovascular or pulmonary condition was documented, cardiac output was not assessed, and the hypotension had already occurred, so the label expresses the susceptibility to recurrence while oral intake was limited and maintenance intravenous fluids had been stopped on day +12 (fluid balance values are not available for this report). The orthostatic hypotension itself is a documented clinical finding.

NANDA-I: Risk for adult falls; risk for imbalanced blood pressure (orthostatic episode).

NIC: Energy management; exercise therapy: ambulation; fall prevention; hypotension management.

NOC: Activity tolerance; fatigue level; fall prevention behavior; vital signs.

### 3.5. Sleep–Rest

Sleep quality was intermittently disrupted by hospital routines, anxiety, and mucositis-related pain. During the peak mucositis period (days +9 through +11), nocturnal pain intensified markedly. Following the initiation of transdermal fentanyl therapy, pain scores and sedation levels were closely monitored, and nursing interventions were clustered to minimize nighttime interruptions. Relaxation and breathing exercise techniques were actively encouraged. At discharge, low-dose melatonin was prescribed, and the patient received comprehensive education on sleep hygiene.

NANDA-I: Ineffective sleep pattern.

NIC: Sleep enhancement; pain management: acute; anxiety reduction.

NOC: Sleep; rest; comfort level.

### 3.6. Cognitive–Perceptual

The patient remained fully oriented and cooperative throughout the hospital stay. Pain assessment was performed at each examination using the Numeric Rating Scale (NRS). No cognitive impairment, delirium, or sensory deficits were observed. Patient education regarding the conditioning regimen, GVHD prophylaxis, laboratory values, and the etiology of specific symptoms was provided in a clear, repetitive manner tailored to the patient’s level of health literacy.

NANDA-I: Acute pain; readiness for enhanced health knowledge.

NIC: Pain management: acute; teaching: prescribed medication; teaching: procedure/treatment.

NOC: Pain control; symptom control; knowledge: treatment regimen.

### 3.7. Self-Perception–Self-Concept

This pattern was particularly salient given the patient’s complex oncological history. Her prior breast cancer and bilateral mastectomy for BRCA mutation had already challenged her body image and sense of physical integrity. Facing AML and allo-HSCT reactivated concerns regarding physical integrity, survival, and her ability to maintain maternal and social roles. Nurses encouraged the expression of feelings about previous cancer experiences and current fears. Changes in appearance during hospitalization (alopecia, fatigue, and central venous catheter use) were openly discussed.

NANDA-I: Risk for situational inadequate self-esteem; excessive fear.

NIC: Emotional support; self-esteem enhancement; coping enhancement.

NOC: Self-esteem; body image; psychological well-being.

### 3.8. Role–Relationship

The patient was married with one child and received strong family support. She experienced role strain due to temporary separation from her child and the inability to perform the usual caregiving responsibilities. Family members were present when allowed and were actively involved in care team communication. Nurses promoted family involvement in the education sessions. Phone and video contact was facilitated when in-person visits were restricted.

NANDA-I: Excessive parental role conflict.

NIC: Family involvement promotion; family support; discharge planning.

NOC: Family coping; caregiver performance; social support.

### 3.9. Sexuality–Reproductive

The patient expressed concerns about long-term hormonal status, menstrual function, and sexual health after allo-HSCT. Nurses provided brief information about the possible gonadal toxicity of myeloablative conditioning and arranged referral to gynecology and reproductive health specialists following hematologic stabilization. Privacy and dignity were maintained throughout all the examinations. The day +15 dysuria and genital burning described in Section 3.3 is also relevant to this pattern. Whether fertility-preservation counseling was offered before induction chemotherapy, which options were discussed, and what the patient chose are not documented in the nursing or epicrisis records available to us, so this report cannot describe them. During the admission, the nursing contribution was limited to assessing her concerns, giving brief information, and arranging referral. The clinic record at 12 months shows a risk-reducing bilateral salpingo-oophorectomy for the BRCA1 variant planned for May 2024, so ovarian function and fertility remained relevant after transplantation. We recommend that fertility counseling be offered and documented before induction for patients of reproductive age, as the ASCO guideline advises [22], and that nurses ask about it during the initial assessment.

NANDA-I: Readiness for enhanced health knowledge (focus: sexual and reproductive health).

NIC: Sexual counseling; teaching: sexuality; referral.

NOC: Knowledge: reproductive health; sexual functioning.

### 3.10. Coping–Stress Tolerance

The transplantation was identified as a significant stressor superimposed on the patient’s prior oncologic experience and chronic autoimmune disease. Initially, the patient engaged in active information-seeking behavior. However, mucositis-related pain, hemorrhoidal bleeding, fever emerging on day +11, and orthostatic hypotension observed on day +16 transiently intensified the patient’s anxiety and fear. Nurses assessed anxiety through direct questioning and provided timely information regarding blood culture results and the engraftment process, explaining that the orthostatic hypotension was most likely engraftment-related and reversible. The patient’s coping process was largely sustained effectively, reinforced by family support and trust in the multidisciplinary team [7].

NANDA-I: Excessive anxiety; excessive fear.

NIC: Anxiety reduction; coping enhancement; emotional support.

NOC: Anxiety level; coping; adaptation to illness.

### 3.11. Value–Belief

The patient emphasized strong family bonds and personal beliefs, which helped her ascribe meaning to her illness and its treatment. She regarded allo-HSCT as both a life-saving opportunity and a test of resilience. Her profound devotion to her child and family reinforced her adherence to treatment. Throughout the care planning process, the patient’s cultural and individual values were respected, and access to spiritual support services was provided.

NANDA-I: Readiness for enhanced spiritual well-being; risk for impaired spiritual well-being.

NIC: Spiritual support; values clarification; decision-making support.

NOC: Spiritual well-being; hope; treatment adherence.

## 4. Outcome and Follow-Up

### 4.1. Clinical Outcomes

Mucositis: Peak BOAS score of 15 on day +7, decreasing to 8 on day +10 and to 5 on day +14 (the physician-recorded grade was 3 on days +9 and +10; see Table 2 footnote). Soft oral intake was resumed by day +10; maintenance intravenous fluids were stopped on day +12.

Pain: Oral pain peaked at NRS 8/10 on day +7, managed with a viscous lidocaine mouth rinse every two hours and systematic oral care. Pain decreased to NRS 4/10 by day +10 and NRS 1/10 by day +14, as mucositis resolved.

Engraftment: Neutrophil engraftment on day +16 without granulocyte colony-stimulating factor administration. Platelet transfusions were administered prophylactically, before counts fell below 20,000/µL. No clinical signs of acute GVHD were observed during hospitalization. The platelet engraftment date, the daily neutrophil count series, and chimerism results are not documented in the available records.

Infection: Febrile neutropenia on day +11 was managed with broad-spectrum antibiotics (meropenem/amikacin), and blood cultures showed no growth. The patient remained hemodynamically stable.

Psychosocial Outcomes: Anxiety was assessed by direct questioning rather than a validated instrument; the patient reported reduced anxiety as engraftment progressed and complications were addressed in a timely manner, and clinical observation was consistent with this. Family coping was supported through educational sessions and communication. At discharge, the patient reported increased confidence in self-management (patient-reported; not formally measured).

Discharge: Day +18 (3 March 2023) with a comprehensive outpatient regimen (proton pump inhibitor, antiviral, trimethoprim–sulfamethoxazole prophylaxis, isoniazid, cyclosporine A, levothyroxine, melatonin, vitamin and magnesium supplementation) and follow-up scheduled for 8 March 2023 (day +23).

Post-Discharge: Gastric neuroendocrine hyperplasia was identified during the post-transplant evaluation (date, method, and clinical relevance are not documented in the available records; the patient was not followed afterward by the transplant unit, so no further information is available to us) and managed according to gastroenterology recommendations. At 12 months (27 February 2024), she was receiving ruxolitinib and tacrolimus; the plan recorded at that visit included stopping tacrolimus and several supportive medications, restarting the vaccination program, and a risk-reducing bilateral salpingo-oophorectomy for the BRCA1 variant planned for May 2024 (the outcome is not documented). The only MRD value documented in the patient’s file is the flow-cytometric bone marrow result of 24 February 2024: 0.56% (CD33+CD34+CD117+ leukemia-associated immunophenotype; limit of detection 0.38%, limit of quantitation 0.56%), as reported by the laboratory. It is given without interpretation, which rests with the treating physicians. At the last available clinic record (18 February 2025), chronic GVHD with liver and ocular involvement was ongoing, and bone marrow reassessment (MRD, NUP98, and chimerism analysis) was planned. Status after February 2025 is not available. Because the patient is no longer followed by the transplant unit, outpatient and physician follow-up (including chimerism, MRD, relapse, survival, late effects, and quality of life) is outside nursing follow-up and is not reported here; follow-up is limited to the clinic records cited above, and status at last contact is as of 18 February 2025. Adherence to oral care protocols was assessed through direct observation and nurse documentation during each shift. The patient consistently performed saline rinses ≥6 times daily from day +4 onward and correctly repeated the infection precautions using teach-back.

Table 4 links each priority diagnosis to the initial assessment, the interventions delivered, and the observed outcome, and states whether the outcome was measured with an instrument or reflects clinical observation or patient report.

### 4.2. Patient Perspective

When allogeneic stem cell transplantation was recommended, a deep fear took hold of me. Yet I regarded it as my most realistic chance at long-term remission. Having previously undergone a prophylactic mastectomy due to my BRCA-positive status, I had a certain degree of experience in making difficult medical decisions and coping with anxiety about the future. But entering this new, grueling process—even as someone who had already endured such challenges—brought on a dimension of uncertainty entirely different from anything I had ever felt before. What weighed on me most heavily were the fears of severe infections, graft-versus-host disease, and the possibility that the disease would return despite intensive treatment.

The conditioning period and the severe mucositis I experienced were the most difficult time I went through. The pain and difficulty swallowing made both eating and speaking unbearable; at times, I felt utterly exhausted, both physically and emotionally. Switching my medications from oral to intravenous administration, starting transdermal pain relievers, and receiving education on oral care—along with knowing that my suffering was recognized and taken seriously, and having nurses constantly around me to help ease it—supported me throughout this process.

During the engraftment process, the fever episodes, sudden dizziness, drops in my blood pressure, and being constantly hooked up to a monitor frightened me deeply, and I thought this ordeal would never end. However, my nurses and my doctor consistently reassured me that these symptoms were common after transplantation, that my fever was normal during this phase, that blood culture monitoring was being performed regularly, and that I was under their close observation. Learning step by step which tests were being done and why, and why antibiotics were started or discontinued, strengthened my confidence in the process and greatly reduced my fear.

I was able to maintain continuous contact with my family through phone and video calls, and this was my greatest source of emotional support in staying resilient. Knowing that they were informed about my condition and that they were closely following the precautions to be taken after my discharge gave me strength. My personal beliefs and my past experience with cancer also played a defining role in this process; especially on nights when my anxiety intensified to an extreme degree, prayer and inner reflection became a vital anchor for me.

When I was discharged, I felt like an entirely different person from the one who had been admitted to the hospital that first day. Although I felt reborn, I was still profoundly exhausted, and I was well aware that this process was ongoing and that recovery would take a long time. My nurses provided me with extensive education—both verbally and in writing—on how to protect myself from infection at home, how and when to take my medications, and which warning signs should prompt me to contact the hospital. The fear that my illness will return still lives within me, because the illness I endured in the past and the challenges I have faced along the way have taken a heavy toll on me. Yet alongside that fear, I also carry optimism, hope, and a deep sense of meaning that comes from having actively participated in a treatment that offered me a genuine chance to move forward.

Note: The term “prophylactic mastectomy” in this section is the patient’s own wording, reproduced verbatim from her account; it is her terminology and not a clinical description of the indication for surgery. The documented clinical history is given in Section 2.3: the hospital records document triple-negative breast cancer in 2021 and a BRCA1 mutation, and bilateral mastectomy because of the BRCA mutation, followed by bilateral breast implants. The records available to us give no further detail on the timing or the surgical indication (for example, whether the surgery was therapeutic, contralateral risk-reducing, or both). The indication for surgery in this report is therefore taken from Section 2.3 and not from the patient’s wording.

## 5. Discussion

This case illustrates that Gordon’s Functional Health Patterns Model can provide a comprehensive and structured framework for organizing nursing assessment and care planning during the allo-HSCT process, integrating physiological and psychosocial domains. Several aspects of this case warrant critical discussion.

First, from the standpoint of physiological management, the course of mucositis (declining from a score of 15 on day 7 to 5 by day 14) and the engraftment timeline (neutrophil recovery after day +16) were consistent with the outcomes expected following myeloablative peripheral blood stem cell transplantation. Busulfan-based conditioning and short-course methotrexate are both recognized contributors to mucositis in this setting. No acute GVHD was observed during hospitalization; acute GVHD is a recognized complication after peripheral blood stem cell transplantation [23]. Prescribing decisions driven by clinical severity—namely, converting oral medications to intravenous administration on day +9, initiating transdermal opioid therapy on day +9, and switching antifungal prophylaxis to micafungin—were made by the physicians. The nursing contribution consisted of systematic assessment, early recognition and reporting of clinical changes, administration of the prescribed treatment, monitoring, education, and evaluation of treatment response; this single case cannot show that the framework itself produced these decisions or the observed clinical course. The BOAS and NRS scores, documented by nurses during care, provided structured metrics for clinical monitoring. Pain peaked at NRS 8/10 on day +7, the day an analgesic mouth rinse was arranged; transdermal fentanyl (25 µg/h) was started on day +9, when grade 3 mucositis was recorded. This interval of about two days with severe pain managed by topical measures is a point for reflection and suggests that earlier criteria for systemic analgesia could be considered; analgesic choices were prescribing decisions. The product labeling restricts transdermal fentanyl to opioid-tolerant patients [24], and posaconazole, a CYP3A4 inhibitor that was restarted on day +13 while the patch was in place (removed on day +14), can raise fentanyl exposure [24]; the records available to us do not document the rationale or a pharmacist review, and these points belong to physician and pharmacist evaluation. The MASCC/ISOO guideline addresses mucositis prevention and management in transplant settings [25]; no photobiomodulation or other preventive measure beyond saline rinses is documented in the available records.

Second, the psychosocial dimensions of this case were unusually complex. The patient’s prior breast cancer and BRCA-related mastectomy had created a preexisting disruption of body image and self-concept, which may have been reactivated by the AML diagnosis and subsequent transplantation. This layered oncologic history necessitates nursing interventions that specifically address the cumulative psychological burden of multiple cancer-related experiences—a need that is not widely emphasized in nursing care reports on allo-HSCT. The explicit inclusion of the self-perception–self-concept, coping–stress tolerance, and value–belief patterns ensured that these needs were assessed within the same care plan as the physiological complications [26]. The anxiety and fear documented in this case are consistent with studies reporting high rates of traumatic stress among patients with acute leukemia undergoing intensive treatment [7].

Third, the model’s structure can strengthen interdisciplinary communication by providing a shared assessment language among nursing, medical, nutrition, and psycho-oncology teams. In this context, Gordon’s 11 patterns were used as a reference and rounding checklist intended to prevent domains from being overlooked during high-risk periods, when physiological urgency can push psychosocial assessment into the background. Structured nurse-led psychosocial support has improved quality of life and mood in patients with advanced cancer [27], although evidence specific to HSCT was not examined in this report. Throughout this process—particularly at the time of diagnosis and thereafter—the presence of professional nurses who employ approaches that reinforce the patient’s trust and strengthen hope, while fostering the development of coping mechanisms, contributes significantly to the patient’s sense of safety. Early recognition by nurses of complications that may arise during the transplantation process, together with the use of standardized checklists in the care of immunocompromised patients, is highly effective in enhancing the quality of care. Therefore, in areas requiring specialized care and training, such as stem cell transplantation, supporting nurses through certification programs to update their knowledge and skills is essential for sustaining high-quality, safe care [28].

Nursing models establish the conceptual framework of care in clinical practice, providing nurses with a systematic roadmap. Practices grounded in these models are generally considered to support a more consistent and organized care process, although the supporting evidence is largely descriptive [29]. In this context, Gordon’s Functional Health Patterns (FHP) model facilitates the detailed collection of patient data across each pattern, allowing the individual’s needs to be identified in a more comprehensive and systematic way. Consequently, nursing care plans can be developed through a higher-quality, holistic approach tailored to the individual’s needs. Indeed, descriptive reports in the relevant literature illustrate how this model can be applied in clinical settings, although they do not establish its effect on patient outcomes [9,10,11,12,29].

Contribution to the literature. Gordon-based nursing case reports have described patients with metastatic breast cancer, metastatic colorectal cancer, depression, and, in one report, chronic myelomonocytic leukemia with type 2 diabetes undergoing HSCT [9,10,11,29]. Compared with that HSCT report [11], this report concerns an allogeneic transplant for AML with a NUP98 rearrangement and prior breast cancer with a BRCA1 mutation. This comparison reflects the author’s reading of the literature and not a systematic database search. This report adds a day-level allogeneic HSCT timeline for AML, named measures (BOAS and NRS) beside the nursing labels, an explicit mapping of the nursing content to Gordon’s patterns, attention to psychosocial, family, reproductive, and spiritual dimensions in a patient with prior breast cancer and a BRCA1 mutation, and the patient’s own perspective. Its main contribution is descriptive: it shows how the framework can organize assessment and care planning in this setting, and it states plainly what was not measured (NOC ratings and validated psychosocial instruments).

This case report has several limitations. As a single case presentation, its generalizability is limited; it cannot be definitively determined whether the outcomes described are attributable solely to the nursing framework or to the overall multidisciplinary care environment. In this report, psychosocial outcomes were assessed primarily on the basis of clinical observation and patient self-report rather than validated psychometric instruments (e.g., the Hospital Anxiety and Depression Scale, FACT-BMT), which limits quantitative comparison with the existing literature. Several elements depend on physician documentation and fall outside the nursing care plan; the physician reports and their detailed results are therefore not included in this nursing report. These include molecular and cytogenetic work-up beyond the results given, measurable residual disease and chimerism, donor selection and familial-variant testing, treatment of the earlier breast cancer (and therefore whether the AML was therapy-related), prescribing rationale and drug interactions (e.g., transdermal fentanyl with posaconazole on days +13 to +14), and fertility-preservation counselling before induction. NOC indicator ratings were not recorded, and weight, albumin, and neutrophil and platelet series were not available for this report. The BOAS value recorded on day +10 (8) and the physician-recorded grade 3 mucositis on the same day are not concordant; they are different instruments and are reported as documented. The grading system used for the physician-recorded mucositis grade (for example, WHO or CTCAE) is not named in the epicrisis or in the clinic records and cannot be established retrospectively; the grades are therefore reported as “grade 2” or “grade 3” as documented and cannot be interpreted against a specific scale. Contraception, menstrual suppression during thrombocytopenia, premature ovarian insufficiency, hormone replacement, and genital GVHD surveillance are not addressed, and no mucositis prevention modality beyond oral care and saline rinses (e.g., photobiomodulation) is documented. Where no formal measurement was made, outcomes are identified in Table 4 as clinical observations or patient-reported experiences. Future studies are recommended to incorporate validated psychosocial measurement tools when applying a structured nursing framework to this patient population. Furthermore, assessment of the sexuality–reproductive pattern remained limited during the acute period; ideally, a more comprehensive reproductive health evaluation should be initiated prior to transplantation. The report also has no comparator, so it cannot show what usual nursing care would have produced; mucositis resolution and engraftment are determined mainly by the conditioning regimen, the graft, and the drugs, and they are not attributed to the nursing framework. Finally, post-discharge information is limited to what the clinic records contain. An isolated MRD result (flow-cytometric bone marrow, 24 February 2024) and documentation of chronic GVHD (ongoing at the last record, 18 February 2025) were available; this is not a comprehensive longitudinal assessment. Serial disease monitoring (MRD, NUP98, and chimerism over time), complete long-term outcomes (relapse, survival, late effects, and quality of life), and the clinical status after February 2025 could not be established. Because the patient is no longer followed by the transplant unit, this report covers the nursing care during transplantation and early recovery and cannot judge the long-term outcome of an AML with a NUP98 rearrangement.

## 6. Conclusions

In this 38-year-old woman undergoing allogeneic HSCT for AML, organizing nursing assessment and care planning around Gordon’s 11 Functional Health Patterns supported systematic attention to both physiological priorities (oral mucositis, infection risk, engraftment-related orthostatic instability) and the psychosocial priorities arising from her layered oncologic history (prior breast cancer with BRCA-related mastectomy, fertility concerns, family role strain) within a single, NANDA-I–NIC–NOC-aligned care plan. The patient achieved neutrophil engraftment, resolution of mucositis, and discharge with structured self-management education, and she reported increased confidence in self-care at discharge. These observations suggest that Gordon’s model can be a useful framework for organizing holistic assessment and care planning during allo-HSCT; whether it improves measurable patient outcomes relative to usual care will require confirmation in larger, comparative studies.

## Figures and Tables

**Table 1:** Baseline clinical and laboratory findings at admission to the transplantation unit.

Reference Range/Comment	Result	Parameter
ABO/Rh typing	A Rh-positive	Blood group
Preserved systolic function (echocardiography)	55–60%	Left ventricular ejection fraction (31 January 2023)
Normal pulmonary function	114% predicted	FEV1 (2 February 2023)
Normal renal function	139.49 mL/min	Glomerular filtration rate (6 February 2023)
Elevated	855 ng/mL	Ferritin
Within normal range	1416 mg/dL	IgG
Low; levothyroxine dose subsequently reduced	0.28 µIU/mL	TSH
Immunity to hepatitis A	Positive (7.43)	Anti-HAV IgG
No acute hepatitis A	Negative	Anti-HAV IgM
Immunity after vaccination or past infection	Positive (22.7)	Anti-HBs
No past hepatitis B infection	Negative	Anti-HBc IgG
No active hepatitis B	Negative	HBsAg
No hepatitis C infection	Negative	Anti-HCV
No HIV infection	Negative	Anti-HIV 1/2 (p24)
Past exposure, immunity	Positive (34.7)	CMV IgG (ELISA)
No acute CMV infection	Negative	CMV IgM (ELISA)
Past exposure, immunity	Positive (27.10)	EBV VCA IgG
No acute EBV infection	Negative	EBV VCA IgM
No past toxoplasmosis	Negative	Toxoplasma IgG
No acute toxoplasmosis	Negative	Toxoplasma IgM
Consistent with latent tuberculosis infection; isoniazid prophylaxis initiated	Positive	Quantiferon-TB (4 February 2023)
Acyclovir prophylaxis initiated	Positive (6.50)	HSV-1 IgM
Acyclovir prophylaxis initiated	Positive (10.50)	HSV-2 IgM
Past immunity; no active infection	IgG positive; IgM negative	Varicella-zoster IgG/IgM

ELISA: Enzyme-linked immunosorbent assay; HAV: Hepatitis A virus; HBsAg: Hepatitis B surface antigen; HBc: Hepatitis B core antigen; HBs: Hepatitis B surface; HCV: Hepatitis C virus; HIV: Human immunodeficiency virus; CMV: Cytomegalovirus; EBV: Epstein–Barr virus; VCA: Viral capsid antigen; TSH: Thyroid-stimulating hormone; FEV1: Forced expiratory volume in 1 s; TB: Tuberculosis.

**Table 2:** Transplant timeline and key clinical events.

Key Events and Clinical Findings	Calendar Date	Day (Relative to HSCT)
Patient admitted to transplant unit in good general condition; baseline workup unremarkable.	6–7 February 2023	−7 to −6
Myeloablative conditioning (busulfan on days −6 to −3, fludarabine on days −6 to −2); stable vital signs, adequate oral intake, no acute complications.	7–10 February 2023	−6 to −3
Last dose of fludarabine administered; patient asymptomatic.	11 February 2023	−2
Cyclosporine A initiated for GVHD prophylaxis; no active complaints.	12 February 2023	−1
Allogeneic peripheral blood stem cell transplantation completed uneventfully after steroid and antihistamine premedication.	13 February 2023	0
Mycophenolate mofetil and methotrexate started; stable condition, no acute toxicity.	14 February 2023	1
Good general condition; adequate oral intake; no fever, no gastrointestinal or respiratory complaints; GVHD prophylaxis continued and cyclosporine A dose adjusted per levels.	15–19 February 2023	+2 to +6
Onset of odynophagia; grade 2 oral mucositis documented; NRS pain score 8/10; Beck Oral Assessment Scale (BOAS) score 15.	20 February 2023	7
Persistent grade 2 oral mucositis; topical treatments intensified; symptomatic relief reported.	21 February 2023	8
Progression to grade 3 oral mucositis; oral medications converted to IV; nutritional supplements and opioid analgesia initiated; antifungal prophylaxis switched to micafungin; hemorrhoidal bleeding managed with platelet transfusion.	22 February 2023	9
Ongoing grade 3 oral mucositis (physician-recorded); BOAS score 8 (nursing assessment; lower than on day +7 although the physician-recorded grade was higher; the two instruments differ and the discordance is acknowledged in the footnote); NRS 4/10 (patient-reported); severe throat pain; first documented fever on night of day +11; broad-spectrum antibiotics (meropenem + amikacin) started; IV fluids and electrolyte replacement adjusted.	23–24 February 2023	+10 to +11
Afebrile under antibiotics; mucositis improving; cyclosporine A continued; maintenance IV fluids stopped.	25 February 2023	12
Oral mucosa improving; posaconazole reintroduced; amikacin discontinued; meropenem continued; patient hemodynamically stable.	26 February 2023	13
BOAS score 5; NRS 1/10; transdermal opioid discontinued; adequate oral intake; no fever.	27 February 2023	14
Oropharyngeal findings largely resolved; complaint of dysuria and genital burning; urinalysis and cultures without significant pathology.	28 February 2023	15
Neutrophil engraftment achieved without G-CSF; orthostatic hypotension managed with IV fluids and methylprednisolone; central venous catheter removed after stabilization.	1 March 2023	16
Orthostatic symptoms resolved; afebrile; meropenem discontinued; continued cyclosporine A and mycophenolate.	2 March 2023	17
Discharge in good clinical condition: progressive clinical improvement; oral intake normalized; mucositis resolved; no signs of acute GVHD; stable hematologic recovery.	3 March 2023	18
Scheduled outpatient follow-up appointment.	8 March 2023	23

HSCT: Hematopoietic stem cell transplantation; GVHD: Graft-versus-host disease; IV: Intravenous; BOAS: Beck Oral Assessment Scale; NRS: Numeric Rating Scale; G-CSF: Granulocyte colony-stimulating factor. Mucositis grade is the physician-recorded clinical grade (the grading scale is not specified in the epicrisis); BOAS is the nursing assessment score; NRS is patient-reported. Scores are reported as documented. The BOAS fell from 15 on day +7 to 8 on day +10 while the physician-recorded grade rose from 2 to 3; this discordance is reported as documented and is not resolved by the available records.

**Table 3:** Comprehensive nursing diagnoses and Gordon pattern-aligned NANDA-NIC-NOC mapping.

Key NIC Interventions and NOC Outcomes	NANDA-I Diagnosis	Pattern
NIC: Teaching: procedure/treatment; Medication management; Infection protection. NOC: Knowledge: infection management; Health-seeking behavior; Infection severity.	Risk for infection; Inadequate health knowledge (transplant process, infection prevention); Readiness for enhanced health self-management	Health perception–health management
NIC: Oral health restoration; Nutrition management; Pain management: acute; Electrolyte management. NOC: Oral mucous membrane integrity; Nutritional status; Pain control; Hydration.	Impaired oral mucous membrane integrity; Inadequate nutritional intake; Risk for impaired water-electrolyte balance	Nutritional–metabolic
NIC: Bowel management; Bleeding precautions; Bleeding reduction; Urinary elimination management; Fluid management. NOC: Elimination status (bowel/urinary); Bleeding severity; Fluid balance.	Risk for excessive bleeding; Risk for impaired intestinal elimination; Impaired tissue integrity (perianal); Impaired urinary elimination	Elimination
NIC: Energy management; Exercise therapy: ambulation; Fall prevention; Hypotension management. NOC: Activity tolerance; Fatigue level; Fall prevention behavior; Vital signs.	Risk for adult falls; Risk for imbalanced blood pressure (orthostatic episode)	Activity–exercise
NIC: Sleep enhancement; Pain management: acute; Anxiety reduction. NOC: Sleep; Rest; Comfort level.	Ineffective sleep pattern	Sleep–rest
NIC: Pain management: acute; Teaching: prescribed medication; Teaching: procedure/treatment. NOC: Pain control; Symptom control; Knowledge: treatment regimen.	Acute pain; Readiness for enhanced health knowledge	Cognitive–perceptual
NIC: Emotional support; Self-esteem enhancement; Coping enhancement. NOC: Self-esteem; Body image; Psychological well-being.	Risk for situational inadequate self-esteem; Excessive fear	Self-perception–self-concept
NIC: Family involvement promotion; Family support; Discharge planning. NOC: Family coping; Caregiver performance; Social support.	Excessive parental role conflict	Role–relationship
NIC: Sexual counseling; Teaching: sexuality; Referral. NOC: Knowledge: reproductive health; Sexual functioning.	Readiness for enhanced health knowledge (focus: sexual and reproductive health)	Sexuality–reproductive
NIC: Anxiety reduction; Coping enhancement; Emotional support. NOC: Anxiety level; Coping; Adaptation to illness.	Excessive anxiety; Excessive fear	Coping–stress tolerance
NIC: Spiritual support; Values clarification; Decision-making support. NOC: Spiritual well-being; Hope; Treatment adherence.	Readiness for enhanced spiritual well-being; Risk for impaired spiritual well-being	Value–belief

NANDA-I: NANDA International; NIC: Nursing Interventions Classification; NOC: Nursing Outcomes Classification. Risk diagnoses reflect the expected treatment-related cytopenias; events that subsequently occurred (e.g., hemorrhoidal bleeding, febrile neutropenia) are reported in Table 2. NOC indicator ratings were not recorded.

**Table 4:** Priority nursing diagnoses linked to assessment, interventions, and outcomes.

Outcome and Type of Evidence	Key Interventions Delivered (NIC)	Defining Characteristics and Initial Assessment	Priority NANDA-I Diagnosis (2024–2026 Label and Code); Related or Risk Factors
BOAS 8 (day +10) and 5 (day +14); NRS 4/10 (day +10) and 1/10 (day +14); physician-recorded oropharynx normal on day +14. Evidence: measured (BOAS nurse-rated, NRS patient-reported).	Oral health restoration (analgesic mouth rinse arranged on day +7, viscous lidocaine rinse every 2 h, saline rinses ≥6 times daily from day +4, intensive oral care); pain management: acute (NRS assessment and reporting; administration and monitoring of prescribed analgesia, namely IV conversion of oral drugs on day +9 and transdermal fentanyl 25 µg/h from day +9).	Odynophagia from day +7 (NRS 8/10; BOAS 15; physician-recorded grade 2); physician-recorded grade 3 by day +9.	Impaired oral mucous membrane integrity (00045) and acute pain (00132). Related factor: mucosal toxicity of busulfan-based conditioning and short-course methotrexate.
Soft oral intake resumed by day +10; maintenance IV fluids stopped on day +12; oral intake normalized by discharge. Evidence: clinical record (weight and albumin values not available for this report).	Nutrition management; nutritional supplement formula from day +9; daily weight, intake–output, and electrolyte monitoring.	Baseline BMI 19.9 kg/m^2^ with adequate intake; severe dysphagia limiting oral intake from day +9.	Inadequate nutritional intake (00343). Related factor: inability to ingest food because of mucositis pain and dysphagia.
Afebrile on day +12; blood culture of 25 February 2023 showed no growth; meropenem discontinued on day +17. Evidence: clinical and laboratory record.	Infection protection and neutropenic precautions; blood culture surveillance; early reporting of fever; administration and monitoring of prescribed empiric meropenem plus amikacin; teach-back on infection precautions.	Expected neutropenia; febrile neutropenia occurred on the night of day +11.	Risk for infection (00004). Risk factors: neutropenia after myeloablative conditioning, immunosuppressive prophylaxis, central venous catheter.
Correctly repeated infection precautions using teach-back; reported increased confidence at discharge. Evidence: direct observation and patient report (no formal knowledge test).	Teaching: procedure/treatment; medication management; structured education reinforced at transitions of care, with teach-back and verbal and written discharge education.	Basic understanding of the diagnosis; inadequate knowledge of conditioning, GVHD prophylaxis, neutropenic precautions, and post-discharge self-management.	Inadequate health knowledge (00435). Related factor: new, complex treatment regimen.
Orthostatic symptoms resolved by day +17. Evidence: clinical observation (repeat blood pressure values not reported).	Hypotension management (blood pressure monitoring in three positions and reporting; administration of prescribed isotonic fluids and methylprednisolone 2 × 40 mg); bed-level mobilization; fall prevention.	Dizziness on day +16; blood pressure 110/69 mmHg supine, 102/69 sitting, and 77/42 standing.	Risk for imbalanced blood pressure (00362). Risk factors: possible inadequate fluid volume (limited oral intake; maintenance IV fluids stopped on day +12; fluid balance values not available for this report); engraftment-related hemodynamic change (physician attribution). This label replaces “risk for decreased cardiac output” (00240), which is defined for individuals with cardiovascular or pulmonary conditions or trauma; none was documented, cardiac output was not assessed, and the hypotension was an observed event.
Patient reported reduced anxiety as engraftment progressed; coping largely sustained. Evidence: clinical observation and patient report (no validated instrument).	Anxiety reduction; coping enhancement; emotional support; timely information on culture results and engraftment; open discussion of appearance changes.	Anxiety and fear identified by direct questioning, transiently intensified by pain, bleeding, fever (day +11), and orthostatic hypotension (day +16); concerns about appearance changes (alopecia, catheter) and prior mastectomy.	Excessive anxiety (00400). Related factor: threat to health status and complications during transplantation.
Continuous family contact maintained; patient described family contact as her greatest source of emotional support. Evidence: patient-reported (Section 4.2).	Family involvement promotion and support; family included in education sessions; phone and video contact when visits were restricted.	Role strain from temporary separation from her child and inability to perform usual caregiving responsibilities (Section 3.8).	Excessive parental role conflict (00387). Related factor: separation from her child because of hospitalization.

NANDA-I: NANDA International (labels and codes follow the 2024–2026 edition [8]); NIC: Nursing Interventions Classification; BOAS: Beck Oral Assessment Scale; NRS: Numeric Rating Scale; GVHD: Graft-versus-host disease. NOC indicator ratings were not recorded. Related and risk factors reflect nursing judgment based on the documented course. Other diagnoses in Table 3 (e.g., ineffective sleep pattern, risk for excessive bleeding) were addressed in care, but their outcomes were not tracked with defined indicators.

## Data Availability

No additional data are available beyond those included in this published article. De-identified clinical documentation is available from the corresponding author on reasonable request and with appropriate ethical approval.

## Abbreviations

The following abbreviations are used in this manuscript:

**Abbreviation**	**Definition**
AML	Acute myeloid leukemia
allo-HSCT	Allogeneic hematopoietic stem cell transplantation
BOAS	Beck Oral Assessment Scale
BRCA	Breast cancer gene
CMV	Cytomegalovirus
CSA	Cyclosporine A
EBV	Epstein–Barr virus
ELISA	Enzyme-linked immunosorbent assay
FAB	French–American–British
FEV1	Forced expiratory volume in 1 s
FHP	Functional health patterns
G-CSF	Granulocyte colony-stimulating factor
GVHD	Graft-versus-host disease
HAV	Hepatitis A virus
HIDAC	High-dose cytarabine
HIV	Human immunodeficiency virus
HLA	Human leukocyte antigen
HSV-1/HSV-2	Herpes simplex virus type 1/2
LDH	Lactate dehydrogenase
MMF	Mycophenolate mofetil
MTX	Methotrexate
NANDA-I	NANDA International
NIC	Nursing Interventions Classification
NOC	Nursing Outcomes Classification
NRS	Numeric Rating Scale
TB	Tuberculosis
WHO	World Health Organization
